# Antifreeze proteins

**DOI:** 10.6026/97320630013400

**Published:** 2017-12-31

**Authors:** Irena Roterman, Mateusz Banach, Leszek Konieczny

**Affiliations:** 1 Department of Bioinformatics and telemedicine, Jagiellonian University - Medical College, Lazarza 16, 31-530 Krakow, Poland; 2 Faculty of Physics, Astronomy and Applied Computer Science - Jagiellonian University, Lojasiewicza 11, 30-348 Krakow, Poland; 3 Chair of Medical Biochemistry, Jagiellonian University - Medical College, Kopernika 7, 31-034 Krakow, Poland

**Keywords:** antifreeze proteins, models, function, activity, ice, anti-ice

## Abstract

The antifreeze protein (AFP) activity is explained using two models. The first model is using ice binding and the second is using antiice structuralization of water molecules. The description of AFP function using anti-ice structuralization of water molecules is less explored. Therefore, it is of interest to explain AFP function using this model. Protein folding is often described using models where hydrophobic residues move away from water getting buried and hydrophilic residues are exposed to the surface. Thus, the 3D Gauss function stretched on the protein molecule describes the hydrophobicity distribution in a protein molecule. Small antifreeze proteins (less than 150 residues) are often represented by structures with hydrophobic core. Large antifreeze proteins (above 200 residues) contain solenoid (modular repeats). The hydrophobic field of solenoid show different distribution with linear propagation of the bands of different hydrophobicity level having high and low hydrophobicity that is propagated parallel to the long axis of solenoid. This specific ordering of hydrophobicity implies water molecules ordering different from ice. We illustrate this phenomenon using two antifreeze proteins to describe the hypothesis.

## Structuralization of water molecules in close neighbourhood of antifreeze proteins

The activity of antifreeze proteins is interpreted as the interaction with water in form of ice similar to protein-ligand docking [1], where the structure of ice appears compatible to the docking areas in antifreeze proteins [2]. The fuzzy oil drop model assuming Gaussian distribution of hydrophobicity in protein molecules treats the surface of molecule as covered by polar groups (lowest hydrophibicity on the surface) [3]. These polar groups influence the order of water molecules in a close neighbourhood (however not limited to one layer). In consequence the water dipoles orientations follow the charge distribution on the protein surface. This conclusion is based on the observation of high accordance of hydrophobicity distribution with the idealized one identified in antifreeze proteins of small size. In large antifreeze proteins the solenoid is present. How this structural form is able to protect against the structuralization of ice? The mechanism is similar. As it is shown in this analysis the solenoid is highly discordant versus the idealized distribution with absence of mono-centric hydrophobic core. Instead of it, bands hydrophobic and hydrophilic in turn propagating linearly, parallel to long axis of solenoid are present. In consequence this form of high differentiation influences water surrounding introducing variable organisation of water molecules in the neighbourhood of protein molecule. This problem was discussed in details in [4]. Here we show two examples of antifreeze proteins.

**Figure 1:**
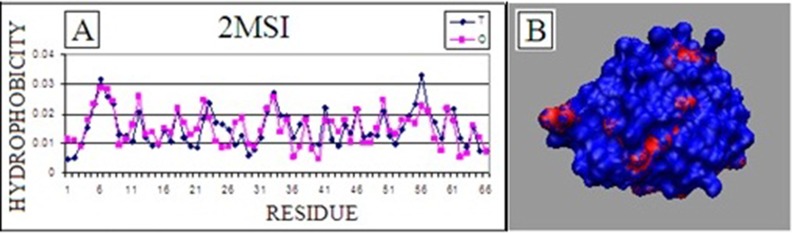
Antifreeze protein isoform of HPLC 12 (PDB ID: 2MSI). (A) T-theoretical (Gaussian) and O-observed distribution of hydrophobicity; (B) 3D representation of hydrophobicity (red) marginally present on the surface in 2MSI

One of them is the small molecule (65 aa) - 2MSI [5]. This protein is of Macrozoarces americanus Ocean pout origin. The second is bigger one - 3VN3 (223 aa in one chain) - fungi origin (Typhula ishikariensis). Its crystal form contains two chains [1]. The fuzzy oil drop model was applied for the analysis of listed proteins. The degree of accordance is expressed by RD parameter, which is equal to 0.377 for this protein. The high accordance can be seen in Figure 1. [3]. In consequence the surface is covered by polar group influencing the structuralization of water molecules in similar way as it is in case of ions, when we use salt (NaCl) in winter time.

**Figure 2:**
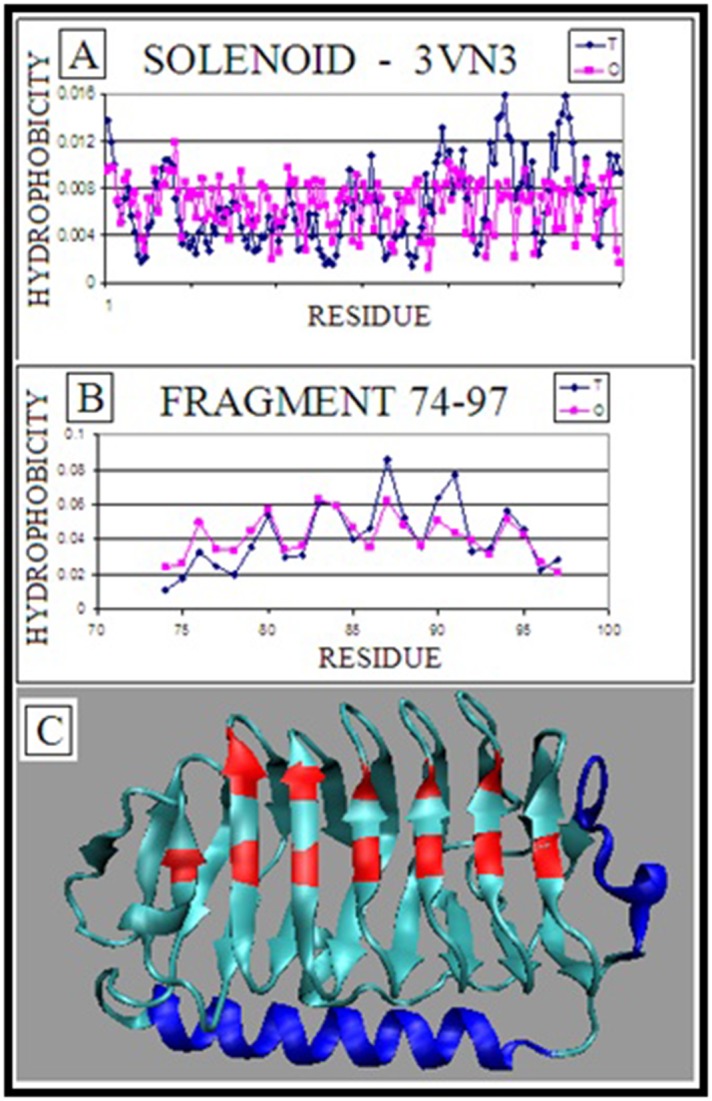
Fungal anti-freeze protein (PDB ID: 3VN3). (A) Expected (T) and observed (O) hydrophobicity distribution in solenoid (modular repeat) part of a fungal antifreeze protein; (B) T and O hydrophobicity distribution in helical part (74-97); (C) 3D representation of 3VN3 with fragments distinguished as blue - high accordance between T and O distribution, red - linear positions of hydrophobic residues in one part of solenoid.

The solenoid super secondary structural form does not follow the mono-centric hydrophobicity distribution RD = 0.736 (Figure 2A). Independently on the position, the distribution is sinusoidlike what can be also seen on 3D presentation (Figure 2C). The linearly ordered positions of highly hydrophobic residues (Figure 2C) those are discordant versus the expected distribution. Fragments locally accordant with expected hydrophobicity distribution - helix (RD=0.469) and C-terminal fragment (RD=0.288) of solenoid (Figure 2B). Their role - probably - is to increase the solubility of the molecule and additionally Cterminal fragment stops the linear propagation protecting the infinite elongation as it is observed in amyloids.

The low hydrophobicity (hydrophilic) band in solenoid influences surrounding water molecules structuralization in the manner following the charge distribution on the protein surface. The band of high hydrophobicity exposed to water environment influences water molecules to order in different way. The contact of water molecules with hydrophobic surface is experimentally observed to be of levitation character [6]. The introduction of such significant differentiation of hydrophobic field in contact with water environment does not support structuralization characteristic for ice. Additionally the high mobility of water molecules is observed on the surface of antifreeze proteins what is accordant with our interpretation of the action of these proteins [7]. The explanation of antifreeze activity of small molecules like saccharides and lipids as docking ice crystals is clearly excluded [8]. Meanwhile fuzzy oil drop model introducing the criteria of water ordering in the neighborhood of antifreeze proteins as well as other small molecules is able to explain the antifreeze activity of molecules of any size.
